# A large deletion within intron 20 sequence of single-copy *PolA1* gene as a useful marker for the speciation in *Oryza* AA-genome species

**DOI:** 10.1270/jsbbs.21075

**Published:** 2022-07-12

**Authors:** Aung Htut Htet, So Makabe, Hiroko Takahashi, Poku Aduse Samuel, Yo-ichiro Sato, Ikuo Nakamura

**Affiliations:** 1 Graduate School of Horticulture, Chiba University, Matsudo, Chiba 271-8510, Japan; 2 BEX Co. Ltd., Itabashi-ku, Tokyo 173-0004, Japan; 3 Kyoto Washoku Institute, Kyoto Prefectural University, Kyoto 606-8522, Japan

**Keywords:** AA-genome species, deletion, *Oryza*, RNA polymerase I largest subunit, single-copy nuclear gene, speciation

## Abstract

*Oryza* AA-genome complex comprises five wild species, *O. rufipogon*, *O. barthii*, *O. longistaminata*, *O. glumaepatula*, and *O. meridionalis*. Evolutionary relationships among these five wild species have remained contentious and inconclusive. We found that intron 20 of *PolA1*, a single-copy nuclear gene, was short (S-type: 141–142 bp) in *O. rufipogon*, *O. barthii*, and *O. glumaepatula*, while long (L-type: *ca.* 1.5 kb) introns were apparent in *O. longistaminata* and *O. meridionalis*. Because *Oryza* species containing BB, CC, EE, FF, and GG genome showed L-type introns, the S-type intron was probably derived from the L-type intron by the deletion of a 1.4 kb fragment through intramolecular homologous recombination between two tandem TTTTGC repeats. Excluding the large deletion sequence, intron 20 sequence of *O. barthii* was identical to that of *O. longistaminata*. As more than 3,470 accessions of *O. rufipogon* and *O. sativa* also contained the same intron 20 sequence with *O. longistaminata* except for single T-nucleotide deletion, which was shared with *O. glumaepatuala*, the deletion of the T-nucleotide probably occurred in the L-type intron 20 of *O. logistaminata*. Deletions of a large 1.4 kb fragment and single T-nucleotide within the intron 20 of *PolA1* gene were considered as useful DNA markers to study the evolutionary relationships among *Oryza* AA-genome species.

## Introduction

The genus *Oryza* (2n = 24 to 48) comprises 24 wild species representing 11 genomes: AA, BB, CC, BBCC, CCDD, EE, FF, GG, HHJJ, HHKK and KKLL. It has two cultivated species, *O. sativa* L. and *O. glaberrrima* Steud, while the other five species: *O. rufipogon* (including *O. nivara*), *O. barthii*, *O. longistaminata*, *O. glumaepatula* and *O. meridionalis* are regarded as wild species in the AA-genome in *Oryza sativa* complex (Ge *et al.* 1999, Vaughan 1994). The wild species of the AA-genome have been recognized as genetic resources for various kinds of useful genes to improve cultivated rice (Jena 2010).

Two cultivated rice species, *O. sativa* in Asia and *O. glaberrima* in Africa, are thought to be originated from *O. rufipogon* and *O. barthii*, respectively (Morishima *et al.* 1992, Oka 1988). Previous study by Second (1985) pointed out that *O. longistaminata* and *O. meridionalis* represented two distinct species. On the other hand, *O. rufipogon*, *O. barthii*, and *O. glumaepatula* were closely related based on isozyme analysis. Many phylogenetic studies of *Oryza* species have been performed with several DNA molecular markers, such as RFLP (Wang *et al.* 1992), RAPD (Ishii *et al.* 1996), AFLP (Aggarwal *et al.* 1999), rDNA spacer (Cordesse *et al.* 1992), transposon (Kanazawa *et al.* 2000), catalase gene (Iwamoto *et al.* 1999), short interspersed element (Motohashi *et al.* 1997), and genome wide sequence analysis (Zhu and Ge 2005); the detailed phylogenetic relationships of AA-genome species are still inconsistent among many studies. Guo and Ge (2005) reported that monophyly in Oryzeae was strongly supported by either individual or combined analyses of both cytoplasmic and nuclear sequence in tribe level. Reconstruction of a phylogenetic tree based on combinations of sequence data from different sources such as plastid, mitochondrial and nuclear DNA produced complicated phylogenetic relationships among wild AA-genome species (Duan *et al.* 2007).

We are interested in the involvement of *PolA1* gene with speciation because it encodes species-specific protein tag sequence not only in plants but also animals, fungi, and protists (Nakamura 2016). The *PolA1*, single-copy nuclear gene, encodes the largest subunit of RNA polymerase I that plays an essential role in 45S rRNA transcription (Seither *et al.* 1997). *PolA1* consists of 21 exons and spans approximately 15.0 kb on chromosome 6 in *Oryza sativa* subsp. *japonica* ‘Nipponbare’ (LOC_Os06g40950 and The Rice Annotation Project Database: Os06g0612200 in Rice Genome Annotation Project, Kawahara *et al.* 2013). Recently, particular DNA sequences from exons 19 to 21 of *PolA1* gene have been useful to elucidate the phylogenetic relationships of *Petunia* (Zhang *et al.* 2008), *Oryza* (Takahashi *et al.* 2009), *Triticum* (Takahashi *et al.* 2010), *Brassica* (Fareed *et al.* 2016) and *Triticum-Aegilops* (Nakamura *et al.* 2009), and *Triticum-Aegilops* and *Hordeum* (Rai *et al.* 2012).

In this study, we found that the intron 20 sequences of *PolA1* gene were differentiated in length into S-type (141–142 bp) or L-type (*ca.* 1.5 kb) in *Oryza* AA-genome species. As *Oryza* species outside the AA-genome had the L-type intron, the S-type intron was probably originated from the L-type intron by the deletion of 1.4 kb DNA fragment. This result suggested good evidence for the evolutionary relationships among *Oryza* AA-genome species.

## Materials and Methods

### Plant materials and DNA extraction

Almost all accessions of *Oryza* species used in this study were provided by the National Institute of Genetics, Japan. Two accessions of *O. longistaminata* were obtained from the Genebank of the International Rice Research Institute (IRRI). Of the 30 accessions, listed in Table 1, of 13 *Oryza* diploid species containing AA, BB, CC, EE, FF, and GG genome were analyzed for the intron 20 sequence of *PolA1* gene. Within AA-genome species, the length of intron 20 was analyzed by PCR in 35 accessions of *O. rufipogon*, 18 accessions of *O. barthii*, 21 accessions of *O. longistaminata*, 17 accessions of *O. glumaepatula*, and 16 accessions of *O. meridionalis* (Supplemental Table 1).

Young leaves (*ca.* 100 mg) of seedlings were frozen in 2-ml plastic tubes with liquid nitrogen and crushed into fine powder using a multi-beads shocker (Yasui Kikai Co., Kyoto, Japan). Total genomic DNA was extracted by CTAB method (Doyle and Doyle 1987) and used for PCR and sequence analyses.

### PCR amplification and direct sequencing

**Fig. 1.**
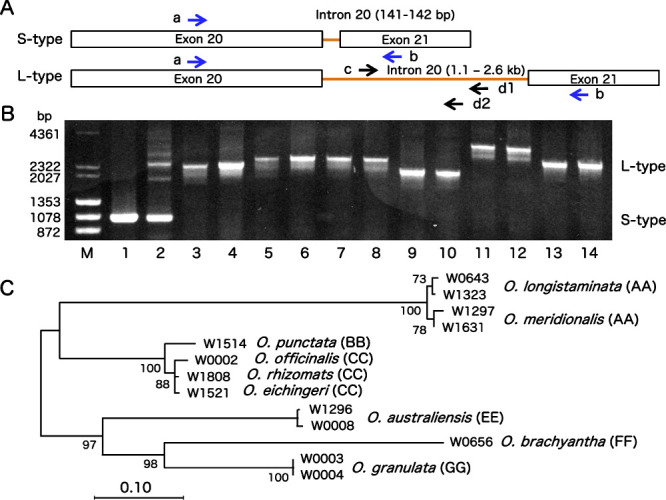
Analysis of intron 20 sequences of *PolA1* genes in *Oryza* diploid species. A: Schematic representation of L-type (1.1–2.6 kb) and S-type (141–142 bp) intron 20 of *PolA1* genes in *Oryza* species. DNA fragments containing the intron 20 sequences were amplified using a pair of primers a and b, which located on the middle of exons 20 and 21 of *PolA1* gene, respectively. Amplified L-type intron 20 sequences were determined using additional sequencing primers c, d1 (AA-genome), and d2 (other than AA-genome). B: PCR products containing the intron 20 sequences in *Oryza* diploid species. M: Size marker λ/*Hin*dIII and φx174/*Hae*III, 1: *O. sativa* Ac221, 2: *O. longistaminata* W0708, 3: *O. punctata* W1514, 4: *O. punctata* W1577, 5: *O. eichingeri* W1521, 6: *O. officinalis* W0002, 7: *O. rhizomztis* W1805, 8: *O. rhizomztis* W1808, 9: *O. australiensis* W1628, 10: *O. australiensis* W1632, 11: *O. brachyantha* W0656, 12: *O. brachyantha* W1706, 13: *O. granulata* W0003, 14: *O. granulata* W0004. C: Neighbor-joining phylogenetic tree was constructed by using MEGA6 software based on the L-type intron 20 sequences in *Oryza* diploid species.

**Fig. 2.**
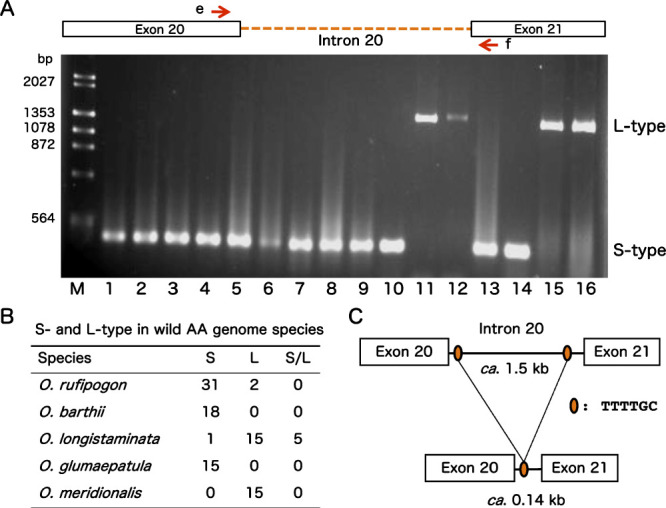
Amplification and variations of intron 20 sequences of *PolA1* genes in AA-genome species. A: DNA fragments containing the intron 20 sequences of AA-genome species were amplified by using a pair of primers e and f, which located on 3ʹ end of exon 20 and 5ʹ end of exon 21, respectively. M: Size marker λ/*Hin*dIII; (*O. rufipogon*) 1: W0106, 2: W1244, 3: W0137, 4: W0630, 5: W0180, 6: W0593, 7: W1230, 8: W2109, (*O. barthii*) 9: W1709, 10: W1643, (*O. longistaminata*) 11: IRGC101198, 12: IRGC101205, (*O. glumaepatula*) 13: W1189, 14: W1192, (*O. meridionalis*) 15: W1625, 16: W1629. B: Variations of L-type and S-type intron 20 of *PolA1* gene in *Oryza* AA-genome wild species. C: S-type intron 20 of *PolA1* gene was probably derived from the L-type intron 20 through the deletion of a large DNA fragment (1.4 kb) by intramolecular homologous recombination between two tandem TTTTGC repeats.

As shown in Figs. 1A and 2A, DNA fragments containing intron 20 (S-type and L-type) were amplified by PCR using two different pairs of primers a and b, and e and f, which were located on the exon 20 and exon 21 of *PolA1* gene, respectively. The primers, listed in Supplemental Table 2, were designed based on the sequence of rice *PolA1* gene (NC_029261, DDBJ). Subsequently, PCR amplification was performed with *Ex*Taq DNA polymerase (TaKaRa, Shiga, Japan) according to manufacturer’s instruction. The PCR conditions were 40 cycles of 94°C for 1 min, 58°C for 1 min for annealing, and 72°C for 2 min for elongation in a PTC200 thermocycler (MJ Research, Waltham, MA, USA).

The amplified PCR products were subjected to 1.0–1.5% agarose gel electrophoresis and purified using a PCR purification kit (QIAquick; Qiagen, CA, USA). DNA sequences of the purified PCR products were determined by direct sequencing with the same primer as used for PCR amplification in an automated DNA sequencer ABI310 (Applied Biosystems, CA, USA) with a Big Dye Terminator Cycle Sequencing kit (Applied Biosystems, USA). Sequences of the L-type intron 20 were determined by using primers c, d1, and d2 as a sequencing primer (Supplemental Table 2). The determined intron 20 sequences of *PolA1* genes in *Oryza* species were registered in the DDBJ as accession nos. (LC638415–LC638446).

### Data analysis

Sequences of PCR products read by direct sequencing were analyzed to determine the positions for donor and acceptor sites of the intron 20 in the *PolA1* gene using NCBI web-based Blast sever (Altschul *et al.* 1990). The sequences were aligned by using CLUSTAW (Thompson *et al.* 1994) and the alignment was then manually adjusted using Genetyx Software ver. 6.0 (Software Development Co., Tokyo, Japan). The phylogenetic tree of intron 20 sequences was constructed using Neighbor-joining method with bootstrap estimate from 1,000 replicates in the MEGA6 software (Tamura *et al.* 2011). We analyzed SNPs in intron 20 of *PolA1* gene between 24412641–24426383 on chromosome 6 of 3,024 accessions of *O. sativa* (http://iric.irri.org/resources/3000-genomes-project) from the International Rice Research Institute (IRRI) and 446 accessions of *O. rufipogon* (http://viewer.shigen.info/oryzagenome21detail/index.xhtml) from the National Institute of Genetics (NIG) to confirm sharing of the same one base T-nucleotide deletion in the S-type intron 20 sequences.

## Results and Discussion

Over the past half century, the utility and potential of various molecular approaches have been effectively used to solve the controversies of evolution and biosystematics that had remained unresolved despite many efforts made through conventional approaches (Avise 1995). Although it is difficult to infer the direction of speciation by comparing association of SNPs and DNA markers, large insertion/deletion inside single-copy conserved gene, such as *PolA1*, are thought to be good markers for determining evolutionary relationships. Previous study by Takahashi *et al.* (2009) reported that PCR products containing intron 19 sequence of *PolA1* gene were differentiated in length among AA, EE, FF and GG genome species in the genus *Oryza* while the amplicon sizes were identical between AA, BB, and CC genome species.

In this study, using a pair of primers a and b (Fig. 1A), amplified DNA fragments containing intron 20 sequences of *PolA1* gene were differentiated into two types, long type (L-type) and short type (S-type), in *Oryza* species. As shown in Fig. 1B, the L-type intron 20 (*ca.* 1.1–2.6 kb) was observed in BB genome species (*O. punctata* W1514, W1577), CC (*O. officinalis* W0002, *O. eichingeri* W1521, *O. rhizomatis* W1808), EE (*O. australiensis* W1628, W1632), FF (*O. brachyantha* W0656, W1706), and GG (*O. granulata* W0003, W0004) (Table 1). Two AA-genome species, *O. sativa* Ac221 showed S-type (141 bp) while *O. longistaminata* W0708 contained both S- and L-type. This result suggested that L-type introns were ancestral to S-type introns and large deletion within the intron 20 happened after the AA-genome species originated.

Within AA-genome species, using a different pair of primers e and f (Fig. 2A), all accessions of *O. rufipogon* showed the S-type intron 20 except two accessions W1235 and W1239 (Fig. 2B, Supplemental Table 1), As Sotowa *et al.* (2013) reported that two New Guinea accessions (W1235 and W1239) shared the same deletions in nuclear genome with *O. meridionalis*, these two accessions were misclassified as *O. rufipogon* (Lam *et al.* 2020). Furthermore, the S-type was also observed in all accessions of *O. barthii* and *O. glumaepatula*. In contrast, all accessions of *O. meridionalis* showed L-type (*ca.* 1.5 kb). In case of *O. longistaminata*, 15 accessions showed L type and one accession W1573 contained S-type. Five accessions had both S- and L-type introns. This result indicated that *O. longistaminata* and *O. meridionalis* predominantly had the L-type whereas *O. rufipogon*, *O. barthii*, and *O. glumaepatula* contained the S-type. Five accessions of *O. longstaminata* having both L-type and S-type were probably hybrids between *O. longistaminata* and *O. barthii*, which shared the same habitat in West Africa.

Although L-type intron 20 sequences of several accessions could not be determined because of sequence heterogeneity (Table 1), Neighbor-joining phylogenetic tree of the L-type sequences in *Oryza* species was constructed (Fig. 1C). Two AA-genome species, *O. longistaminata* and *O. meridionalis*, had closely related L-type sequences. *Oryza punctata* (BB-genome) and *O. officinalis*, *O. eichingeri*, *O. rhizomatis* (CC-genome species) formed a single clade, which was closely related to that of AA-genome species. *Oryza australiensis* (EE), *O. brachyantha* (FF), and *O. granulata* (GG) formed paraphyletic groups those were distantly related to AA-genome species.

The phylogenetic analyses based on the L-type intron 20 sequence (Fig. 1C) was consistent with those based on nuclear ribosomal DNA sequence (Kim *et al.* 2015) and multiple SINE inserts (Cheng *et al.* 2002), which supported the position of *O. longistaminata* as the basal AA-genome species. The ancestor of the Asian *Oryza* AA-genome species was diverged from ancestor of *O. longistaminata* in Africa involving the changes from perennial to annual and sympatric speciation during the course of evolution (Cheng *et al.* 2002, Iwamoto *et al.* 1999, Ohtsubo *et al.* 2004)

**Fig. 3.**
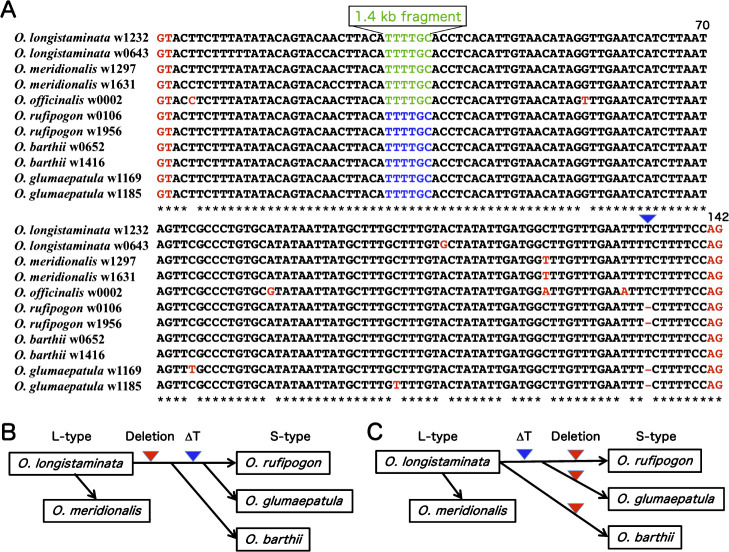
Alignment of intron 20 sequences of *PolA1* gene in *Oryza* AA-genome species. A: The intron 20 sequences excluding 1.4 kb fragments at TTTTGC (green letter) were shown in *O. longistaminata*, *O. meridionalis*, and *O. officinalis* (CC genome). And intron 20 sequences containing single copy of TTTTGC (blue) were shown in *O. rufipogon*, *O. barthii*, and *O. glumaepatula*. Single nucleotide polymorphism (SNP) and donor/acceptor sites at both ends of intron 20 were shown in red letter. Insertion/deletion of T-nucleotide was shown by blue triangle. B: A proposed evolutionary relationships among *Oryza* AA-genome species. Scenario 1: deletion of 1.4 kb fragment in the intron 20 (red triangle) first happened in *O. longistaminata* when *O. barthii* originated. Then, single T-nucleotide deletion occurred in the intron 20 (blue triangle) when common ancestral species of *O. rufipogon* and *O. glumaepatula* originated. *O. meridionals* probably originated from *O. longistaminata*. C: Scenario 2: single T-nucleotide deletion first happened in an accession of *O. longistaminata* which was common ancestral to *O. rufipogon* and *O. glumaepatula*. Deletions of the same 1.4 kb fragment independently happened in the intron 20 during the speciation from *O. longistaminata* to *O. barthii* and from *O. longistaminata*-like species to *O. rufipogon* and *O. meridonalis*.

We found that a large DNA fragment (*ca.* 1.4 kb) was probably deleted between two tandem TTTTGC repeats in the L-type intron, which resulted in the S-type intron (141–142 bp) (Fig. 2C). Also, the identical two tandem repeats were present at the same positions in the L-type intron of *O. officinalis* W0002 (Fig. 3A). Excluding the large 1.4 kb sequence, sequences of S- and L-type introns were highly homologous (Fig. 3A). In detail, intron 20 sequence of *O. barthii* W0652 and W1416 was identical to that of *O. longistaminata* W1232. And both annual W0106 (*O. nivara*) and perennial W1956 accessions of *O. rufipogon* as well as template *japonica* ‘Nipponbare’, tropical *japonica* Ac221, and *indica* Ac130 of *O. sativa* contained the same intron 20 sequence with *O. longistaminata* W1232 except for single T-nucleotide deletion (Fig. 3A). Interestingly, the same single T-nucleotide deletion was shared with two accessions (W1169, W1185) of *O. glumaepatula*.

The evolutionary relationships among *O. rufipogon*, *O. barthii*, *O. longistaminata*, *O. glumaepatula* and *O. meridionalis* have long been a subject of controversy (Vaughan *et al.* 2005, Wang *et al.* 1992, Zhu and Ge 2005). Based on whole genome sequencing of 3,024 accessions of *O. sativa* (IRRI 3K SNP project) and 446 accessions of *O. rufipogon* (Oryza base, NIG), *O. rufipogon* and *O. sativa* shared the same one base T-deletion in the S-type intron 20 sequences (Fig. 3A), because there was no SNP in the intron 20 between columns BF and BG in Supplemental Table 3 (*O. sativa*) and columns FJ and FL in Supplemental Table 4 (*O. rufipogon*). While *O. longistaminata* and *O. barthii* shared the non T-deletion in their intron 20.

These data proposed two scenarios of evolutionary relationships among *Oryza* AA-genome species. Scenario 1: a large deletion of 1.4 kb fragment in the intron 20 first happened in an accession of *O. longistaminata* when *O. barthii* originated. Then, single T-nucleotide deletion occurred in the intron 20 when common ancestral species of *O. rufipogon* and *O. glumaepatula* originated (Fig. 3B). Also, annual species *O. meridionalis* in Australia had been probably originated from *O. longistaminata*. Scenario 2: single T-nucleotide deletion first happened in the L-type intron 20 of an accession in *O. longistaminata* which was common ancestral to *O. rufipogon* and *O. glumaepatula* (Fig. 3C). Then, deletions of the same 1.4 kb fragment independently arose during the speciation from *O. longistaminata* to *O. barthii* and from a *O. longistaminata*-like species to *O. rufipogon* and *O. meridonalis*. Extensive sequence analysis of the intron 20 in *PolA1* gene will be necessary in AA-genome species except for *O. rufipogon* and *O. sativa* to reveal which of two scenario will be correct.

A previous study by Akimoto *et al.* (1997) presumed that *O. glumaepatula* was associated both of *O. rufipogon* and *O. longistamianta*. In this study, as *O*. *glumaepatula* shared the S-type intron 20 sequence containing single T-nucleotide deletion with *O. rufipogon* (Fig. 3A), it might be originated with relation to *O. rufipogon* (Vaughan *et al.* 2005, Yin *et al.* 2015). Two cultivated species, *O. sativa* and *O. glaberrima*, were independently originated from *O. rufipogon* in South-east Asia and *O. barthii* in West Africa, respectively. The origins of two cultigens have been supported by many researches (Aggarwal *et al.* 1999, Chang 1976, Zhu and Ge 2005).

In this study, we found that intron 20 sequences of the *PolA1* gene differed by an order of magnitude in length between two L-type (*ca.* 1.5 kb) species *O. longistaminata* and *O. meridionalis* and three S-type (141–142 bp) species *O. rufipogon*, *O. barthii*, and *O. glumaepatula*. Therefore, three S-type species were derived from ancestral *O. longistaminata* in South Asia (*O. rufipogon*), West Africa (*O. barthii*) and South America (*O. glumaepatula*). The annual species *O. meridionalis* was also originated from perennial species *O. longistaminata* in Australia. Although the mechanism underlying the speciation of these new species from *O. longistaminata* remains to be resolved, ancestral *O. longistaminata* accessions except Africa were displaced over time by newly arisen species. These findings could potentially suggest that *O. longistaminata* was used to distribute in the past not only in Africa but also in Asia and Australia.

## Author Contribution Statement

IN and YS planned this study. HAH, SM, HT and PS performed molecular and phylogenetic analyses. HAH and IN wrote manuscript.

## Supplementary Material

Supplemental Table 1, 2

Supplemental Table 3

Supplemental Table 4

## Figures and Tables

**Table 1.** Materials to analyze the intron 20 sequence of *PolA1* gene in *Oryza* species

Speices	Accession	Description	Intron 20	Genome
*O. sativa*	‘Nipponbare’	Temperate Japonica	141 bp	AA
	Ac221	Tropical Japonica	141 bp	AA
	Ac130	Indica	141 bp	AA
*O. rufipogon*	W0106	Annual, India	141 bp	AA
	W0107	Annual, India	141 bp	AA
	W1724	Perennial, India	141 bp	AA
	W1956	Perennial, China	141 bp	AA
*O. barthii*	W0652	Annual, Sierra Leone	142 bp	AA
	W1416	Annual, Sierra Leone	142 bp	AA
*O. longistaminata*	W0643	Perennial, Gambia	1499 bp	AA
	W0708	Perennial, Guinea	n.d.	AA
	W1232	Perennial, Tanganyika	1523 bp	AA
*O. meridionalis*	W1297	Annual, Australia	1519 bp	AA
	W1631	Annual, Australia	1523 bp	AA
*O. glumaepatula*	W1169	Perennial, Cuba	141 bp	AA
	W1185	Perennial, Suriname	141 bp	AA
*O. punctata*	W1514	Kenya	1485 bp	BB
	W1577	Nigeria	n.d.	BB
*O. officinalis*	W0002	Thailand	1764 bp	CC
	W0614	Burma	n.d.	CC
*O. eichingeri*	W1521	Uganda	1767 bp	CC
	W1527	Uganda	n.d.	CC
*O. rhizomatis*	W1805	Sri Lanka	n.d.	CC
	W1808	Sri Lanka	1766 bp	CC
*O. australiensis*	W0008	Australia	1103 bp	EE
	W1296	Australia	1100 bp	EE
*O. brachyantha*	W0656	Guinea	2643 bp	FF
	W1401	Sierra Leone	n.d.	FF
*O. granulata*	W0003	India	1634 bp	GG
	W0004	India	1634 bp	GG

n.d.: sequence was not determined because of sequence heterogeneity.
