# Supplementary material for: A large deletion within intron 20 sequence of single-copy PolA1 gene as a useful marker for the speciation in Oryza AA-genome species
Source: Breed Sci. 2022 Jul 12;72(3):267–73. doi: 10.1270/jsbbs.21075 (PMC9653197; doi:10.1270/jsbbs.21075)
Supplement: Supplementary file 1 — Supplemental Table 1, 2 [file 72_267_s1.pdf]

doi: 10.1270/jsbbs.21075

Supplemental Table 1. Wild rice strains used in this study

Species	Accession	Origin	Type
O. sativa	'Nipponbare'	Temperate japonica	S
	Ac221	Tropical japonica	S
	Ac130	indica	S
O. rufipogon	W0106	India	S
	W0107	India	S
	W0108	India	S
	W0137	India	S
	W0180	Thailand	S
	W0593	Malaysia	S
	W0610	Burma	S
	W0630	Burma	S
	W1230	Indonesia	S
	W1235	New Guinea	L
	W1236	New Guinea	S
	W1238	New Guinea	S
	W1239	New Guinea	L
	W1244	Nepal	S
	W1551	Thailand	S
	W1666	India	S
	W1669	India	S
	W1681	India	S
	W1685	India	S
	W1690	Thailand	S
	W1715	China	S
	W1724	China	S
	W1852	Thailand	S
	W1807	Sri Lanka	S
	W1865	Thailand	S
	W1866	Thailand	S
	W1939	Thailand	S
	W1945		S
	W1981	Indonesia	S
	W1983	Indica	S
	W2014	India	S
	W2078	Australia	S
	W2109	Australia	S
	W2263	Cambodia	S
	W2266	Laos	S
O. barthii	W0042	West Africa	S
	W0652	Sierra Leone	S
	W0698	Guinea	S
	W0720	Mali	S
	W0747	Mali	S
	W1050	Gambia	S
	W1063	Gambia	S
	W1410	Sierra Leone	S
	W1416	Sierra Leone	S
	W1473	Chad	S
	W1574	Nigeria	S
	W1588	Cameroun	S
	W1605	Nigeria	S
	W1642	Botswana	S
	W1643	Botswana	S
	W1646	Tanzania	S
	W1702	Mali	S
	W1709		S
O. longistaminata	W0643	Gambia	L
	W0708	Guinea	L/S
	W1004	Ghana	L
	W1232	Tanganyika	L
	W1413	Sierra Leone	L/S
	W1420	Mali	L
	W1423	Mali	L/S
	W1444	Ivory Coast	L/S
	W1448	Ivory Coast	L
	W1454 (B)	Burkina Faso	L
	W1460	Benin	L
	W1465	Nigeria	L
	W1504	Tanganyika	L
	W1508	Madagascar	L
	W1540	Congo	L
	W1570	Nigeria	L
	W1573	Nigeria	S
	W1624	Cameroun	L/S

Supplemental Table 1. Wild rice strains used in this study

	W1650	Tanzania	L
	IRGC101198	(IRRI)	L
	IRGC101205	(IRRI)	L
O. glumaepatula	W1169	Cuba	S
	W1171	Cuba	S
	W1183	Guyana	S
	W1185	Suriname	S
	W1186		S
	W1187	Brazil	S
	W1189	Brazil	S
	W1191	Brazil	S
	W1192	Brazil	S
	W1196	Colombia	S
	W1477	Brazil	S
	W2140	Brazil	S
	W2145	Brazil	S
	W2149	Brazil	S
	W2160	Brazil	S
	W2165	Brazil	S
	W2199	Brazil	S
O. meridionalis	W1297	Australia	L
	W1299	Australia	L
	W1300	Australia	L
	W1625	Australia	L
	W1627	Australia	L
	W1629	Australia	L
	W1631	Australia	L
	W1635	Australia	L
	W1638	Australia	L
	W2069	Australia	L
	W2071	Australia	L
	W2077	Australia	L
	W2079	Australia	L
	W2081	Australia	L
	W2103	Australia	L
	W2105	Australia	L

Supplemental Table 2. Primers used in this study

Name	Sequence
a	5'-CCTTGAGAACTGTTTTTATTGATG-3'
b	5'-TGAAATCCGCAATCAAGTTCAGATG-3'
c	5'-GGATTGGCACCATTGGAAACGCAG-3'
d1	5'-ACAACTTCTCCACCAACATTCTCT-3'
d2	5'-TAATCTAATGGTCACTAAAATAAG-3'
e	5'-GAGCAACCTCATATTCTGTTAGCC-3'
f	5'-CTTACAGGCCTTGACAAAAACAGA-3'
